# Neuromodulation Interventions for Language Deficits in Alzheimer’s Disease: Update on Current Practice and Future Developments

**DOI:** 10.3390/brainsci15070754

**Published:** 2025-07-16

**Authors:** Fei Chen, Yuyan Nie, Chen Kuang

**Affiliations:** School of Foreign Languages, Hunan University, Lushannan Road No. 2, Yuelu District, Changsha 410082, China; chenfeianthony@gmail.com (F.C.); kuangchen@hnu.edu.cn (C.K.)

**Keywords:** neuromodulation, Alzheimer’s disease, language, rehabilitation, rTMS, tDCS, DBS

## Abstract

Alzheimer’s disease (AD) is a leading cause of dementia, characterized by progressive cognitive and language impairments that significantly impact communication and quality of life. Neuromodulation techniques, including repetitive transcranial magnetic stimulation (rTMS), transcranial direct current stimulation (tDCS), and deep brain stimulation (DBS), have emerged as promising interventions. This study employs bibliometric analysis to evaluate global research trends in neuromodulation treatments for AD-related language impairments. A total of 88 publications from the Web of Science Core Collection (2006–2024) were analyzed using bibliometric methods. Key indicators such as publication trends, citation patterns, collaboration networks, and research themes were examined to map the intellectual landscape of this field. The analysis identified 580 authors across 65 journals, with an average of 34.82 citations per article. Nearly half of the publications were produced after 2021, indicating rapid recent growth. The findings highlight a predominant focus on non-invasive neuromodulation methods, particularly rTMS and tDCS, within neurosciences and neurology. While research activity is increasing, significant challenges persist, including ethical concerns, operational constraints, and the translational gap between research and clinical applications. This study provides insights into the current research landscape and future directions for neuromodulation in AD-related language impairments. The results emphasize the need for novel neuromodulation techniques and interdisciplinary collaboration to enhance therapeutic efficacy and clinical integration.

## 1. Introduction

Alzheimer’s disease (AD) has been identified as the most prevalent type of dementia among the elderly population [1]. With over 50 million people currently living with dementia—a number projected to triple by 2050—AD is increasing in prevalence at a rate disproportionate to longevity, placing a significant burden on individuals, families, healthcare systems, and the global economy [2,3]. Characterized by degenerative changes across multiple neurotransmitter systems, AD can be associated with clinical impairments in various cognitive domains, including attention, reasoning, memory, and executive function [4]. Concomitantly with these symptomatic variants, dysfunction in speech and language abilities has been recognized as a prominent marker in the early stages of AD [5,6], and serves as a key indicator for detecting mild cognitive decline [7].

Loss of certain facets of language occurs in a significant proportion of AD patients and deteriorates with progression of the illness to cause significant disability [8,9,10]. Generally, AD patients exhibit a mixture of expressive and receptive language deficits, encompassing naming difficulties, compromised auditory and reading comprehension, disfluent and content-devoid speech, and semantic paraphasia [10,11,12,13]. In a significant proportion of cases, the language deficits associated with AD are evident in various aphasia phenotypes [14,15]. The severity of language impairments in AD is closely tied to metabolic changes and atrophy across multiple cortical regions [16,17]. Key areas in the left hemisphere are particularly involved in different aspects of language processing, including semantic representation and word comprehension [16], lexical retrieval and naming ability [18], as well as fluency and executive aspects of language function [16,19,20]. This network-based model is further supported by research on logopenic progressive aphasia (LPA), a subtype of primary progressive aphasia (PPA) associated with AD pathology. LPA is characterized by atrophy in the left temporoparietal junction, which disrupts both phonological processing and sentence repetition, reinforcing the idea that AD-related language deficits stem from widespread degeneration across semantic, phonological, and executive language networks [14,15,21].

The conventional approach to addressing AD encompasses both pharmacological interventions and behavioral strategies (i.e., cognitive rehabilitation, holistic techniques, brief psychotherapy and alternative strategies) [22]. However, pharmacological treatments merely serve to control AD symptoms, rather than alter the progression of the disease. Behavioral therapy is often limited by inadequate resources and infrastructure in acute care settings [23], while its effectiveness diminishes in advanced stages due to progressive neurodegeneration and individual variability in treatment response [24]. Furthermore, the neural mechanisms underlying the beneficial effects of behavioral interventions remain largely unknown.

Over the past decade, neuromodulation techniques have made impressive advances in the treatment of neuropsychiatric diseases [25,26]. Deep brain stimulation (DBS) is a technique that involves the surgical implantation of a lead to deliver focal electrical modulation to specific neural circuits within the brain. This method targets particular brain areas of interest, facilitating the modulation of neural network activity [27]. Seminal studies on DBS in AD have indicated several beneficial effects of this treatment, including a reduction in cognitive decline and hippocampal atrophy, as well as enhancements in cerebral glucose metabolism and brain connectivity among AD patients [28,29,30,31]. However, evidence for the use of DBS to treat dementia, especially for related language impairments, is preliminary and limited [32]. Another invasive technique, vagus nerve stimulation (VNS), represents a Food and Drug Administration (FDA)-approved therapeutic intervention for the reduction of intractable neuropsychiatric conditions [33,34]. The vagal nerve plays a pivotal role in the parasympathetic nervous system, serving as a conduit for extensive sensing and signaling to and from visceral organs. It exerts a crucial modulatory influence within the central nervous system [33]. In the realm of AD treatment, several studies emphasize the impact of VNS on the cognitive and memory function in patients [35,36].

Furthermore, non-invasive transcranial stimulation techniques have been increasingly utilized to enhance cognitive performance in patients with neurological conditions [37]. Encouraging outcomes in adult neuropsychiatric disorders have stimulated active investigation into non-invasive neuromodulation methodologies in pediatric and adolescent populations, particularly transcranial magnetic stimulation (TMS) and transcranial direct current stimulation (tDCS) [38]. TMS is a technique that administers a series of magnetic pulses in rapid succession, achieving frequencies of up to 100 Hz [39]. This technique is well characterized in the context of single-pulse and paired-pulse stimulation of the primary motor cortex. When applied as repetitive transcranial magnetic stimulation (rTMS) in trains of pulses, it typically disrupts specific cognitive operations that are presumably mediated by the stimulated cortical region during the stimulation period [40]. Numerous studies have shown that rTMS is a promising approach for cognitive enhancement, resulting in improved perceptual discrimination, motor learning, accelerated eye movements, expedited visual searching, and enhanced object identification [41]. Additionally, the clinical application of rTMS has been associated with superior performance on tasks related to attention, memory, and language [41]. Specifically, several investigations have demonstrated that rTMS has lasting beneficial effects on language performance in patients with AD, particularly in tasks involving action naming and sentence comprehension [39,42,43]. These cognitive and linguistic improvements are believed to arise from the modulation of synaptic plasticity, primarily through mechanisms of long-term potentiation (LTP) and long-term depression (LTD). High-frequency rTMS may induce LTP-like effects by strengthening synaptic connections, while low-frequency stimulation may trigger LTD-like processes, downregulating maladaptive pathways [44]. In the context of AD, where synaptic dysfunction is a hallmark, these neurobiological mechanisms are critical in restoring or compensating for disrupted neural networks. Through the modulation of cortical excitability and the rebalancing of network dynamics, rTMS can augment functional connectivity within language-related regions, thereby facilitating improved clinical outcomes.

The technique of tDCS, as another non-invasive neuromodulation method, is regarded as a potential approach to the rehabilitation of neurodevelopmental disorders as well. This technique modulates spontaneous neuronal activity by delivering a weak direct current to the scalp, which induces prolonged functional after-effects in the brain [45]. Studies have demonstrated that tDCS applied to the left dorsolateral prefrontal cortex, the left posterior perisylvian region, and Broca’s area significantly enhances daily language abilities and specific language performance in dementia patients with primary progressive aphasia (PPA) [46,47]. Notable improvements have been observed in tasks involving language repetition, reading, picture naming, and auditory comprehension [47].

Rapid advancements in neuromodulation techniques, coupled with their promising potential in medical applications, are transforming the field of neuroscience. In recent years, there has been a significant surge in research activity involving AD. This trend underscores the necessity for a comprehensive examination of the current research landscape and its developmental structure to guide future investigations in this domain. Bibliometric analysis, utilizing mathematical and statistical methods, provides a robust framework for evaluating a large body of literature, allowing researchers to map the intellectual structure of this evolving discipline. This methodology has been effectively employed to analyze various fields, including neurorehabilitation [48], neurodegenerative diseases [49], and speech, language, and hearing sciences [50]. By systematically examining citation counts, collaboration networks, and keyword analyses, bibliometric studies can elucidate the trajectory of research in a particular topic area. Such analyses not only highlight key contributors such as journals, institutions, and countries, but also pave the way for informed clinical recommendations and future investigations in this critical area of study [34,51,52,53]. In light of the rapidly expanding literature on neuromodulation treatments for language impairment in AD patients, a bibliometric analysis is of paramount importance to assist researchers in this field in gaining an informed understanding of the evolving research landscape and fostering international and interdisciplinary collaboration [54]. However, to the best of our knowledge, there has been no bibliometric analysis investigating the application of neuromodulation techniques for language impairment in AD to date. Accordingly, the present study employs bibliometrics and literature visualization tools to analyze the global status of research into neuromodulation techniques for the treatment of language impairment in AD. The results are presented in the form of a visual map, which facilitates further analysis of research hotspots, future trends, and application prospects of neuromodulation techniques in AD-related language deficits.

## 2. Methods

The present bibliometric review was conducted according to the methodology and work flow adopted by Xiao et al. [34] and Zhang et al. [53]. First, we collected the bibliometric data through a systematic literature search. Subsequently, an in-depth evaluation of the selected data was performed, encompassing a comprehensive assessment of the research field and a co-word analysis. The results incorporated a number of variables, including publication trends, the most cited journals, studies, authors, collaborative networks, and relationships. Based on these analyses, we discuss the current state of research as well as comparing the utilization of neuromodulation approaches in AD regarding language to their uses in other disciplines and aspects.

### 2.1. Search Strategy

Records of all relevant publications were retrieved from the core collection of Web of Science Core Collection (WoSCC). The main topics of data retrieval were identified from preliminary bibliometric studies and literature reviews, focusing on three areas: “Alzheimer’s Disease (AD)”, “neuromodulation techniques”, and “language”. We applied an advanced search strategy of topic search, which was described as follows: TS = (“Alzheimer” OR TS = “AD” OR TS = “dementia” OR TS = “cognitive disorder” OR TS = “memory disorder”) AND TS = (“neuromodulation techniques” OR TS = “deep brain stimulation” OR TS = “DBS” OR TS = “vagus nerve stimulation” OR TS = “VNS” OR TS = “transcranial direct current stimulation” OR TS = “tDCS” OR TS = “transcranial magnetic stimulation” OR TS = “TMS” OR TS = “transcranial ultrasound stimulation” OR TS = “TUS”) AND TS = (“language” OR TS = “speech”). The literature was searched and obtained independently by two authors before comparison of their findings. Any discrepancies were resolved through consultation or by seeking the opinion of a third specialist to achieve a consensus. The publication date of all retrieved documents ranged from 1 January 2000 to 31 December 2024.

### 2.2. Screening Strategy

The selection of publications was conducted in a cautiously implemented manner and performed on a single day, 5 February 2025, in order to circumvent any potential biases that might be introduced by daily updating of the database. We included a variety of document types including journal articles, conference papers, review articles, early-access articles and letters, and those of the following types were excluded: letters, notes, books, book chapters, meeting abstracts, corrections, editorial materials, and retracted articles. Non-English language articles and publications with an English title and abstract were also encompassed by this bibliometric analysis. Ultimately, a total of 441 publications were obtained from the WoSCC database and selected for manual screening.

**Figure 1:**
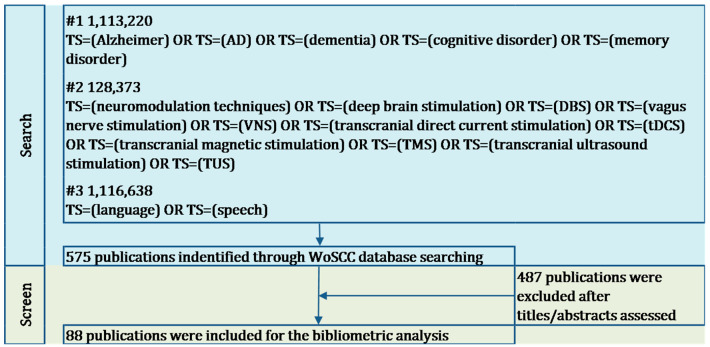
Methodological flowchart for bibliometric review.

The publications subjected to manual screening were restricted to those meeting three specific criteria: (1) focusing on AD; (2) employing neuromodulation techniques as a treatment; and (3) involving investigation of the language ability of participants. Two authors (YN and CK) firstly classified all records into include, exclude, and pending based on their titles and abstracts, in accordance with the established criteria discussed previously. Pending publications were then checked by another researcher and discussed regarding inclusion or exclusion by three researchers together. The full text was consulted when necessary. Finally, 88 publications were included for further analysis. The entire retrieval process is presented in Figure 1.

### 2.3. Data Extraction

The bibliometric data pertaining to each pertinent publication was duly extracted, encompassing authors, affiliations, article titles, document types, journal names or conference names, abstracts, keywords, number of times cited, and cited references. A manual examination was conducted to ensure the accurate supplementation of all absent items.

### 2.4. Statistical Analysis

The open-source bibliometrix R-package (https://www.bibliometrix.org/home/, accessed on 5 February 2025 ) was applied to analyze and visualize the bibliographic data from WoSCC [55].

For descriptive analysis, the coefficients of Lotka’s Law were calculated for the dataset by analyzing the number of authors and their corresponding publication frequencies. As a bibliometric measure of scientific productivity and authorship concentration, Lotka’s Law postulates that the number of authors publishing a specific number of papers is inversely proportional to the square of the number of publications. In particular, a relatively small group of highly prolific authors is responsible for the majority of publications, while the majority of authors publish only a single paper. The formula of Lotka’s law, which relates the number of authors (*y*) to the number of contributions (*x*), is expressed as

$$x^{\beta }y=C$$

where *y* is the number of authors contributing *x* publications, and *C* is a constant specific to the field. *β* represents the exponent that defines the rate of decline in the number of authors as the number of their publications increases. In this case, a higher *β* value indicates a greater concentration of authorship, whereas a lower value suggests the absence of a dedicated group of authors in a given research field [56].

These coefficients displayed the publication trends and identified the most relevant publication outlets and institutes as well as the most impactful articles, thus enabling the identification of patterns of productivity and the extent of authorship concentration in the field under study.

In order to quantify the impact of the publications in question, we employed two citation metrics: global citations and local citations. Global citations quantify the number of times an article has been referenced by other articles within the databases from which the collection has been downloaded. Local citations, on the other hand, indicate the number of times the study has been referenced by the other 67 studies within the same analyzed collection.

For the network analysis of country collaborations, we opted for the Walktrap clustering algorithm due to its effectiveness in producing high-quality partitions and its efficiency in handling large networks [56,57]. To investigate the links between publication outlets, research topics, and countries, we generated a three-field plot using a Sankey diagram, which illustrated the relationships among leading publication sources, key topics, and countries.

We conducted a co-word analysis to identify the frequency of publications that simultaneously include two specific keywords [58]. To visualize the conceptual framework of the research field, we applied the Fruchterman–Reingold layout [59], and used the Louvain clustering algorithm to detect clusters representing shared concepts [60]. The association strength measure was used to normalize the keyword co-occurrences [61].

We then generated a series of strategic maps, where each cluster or theme was positioned within a specific quadrant based on its Callon’s centrality and density values [62]. In these strategic diagrams, the horizontal axis represents centrality, which measures the extent of a cluster’s connections with other clusters in the network, indicating the significance of the research theme in the field [63]. The vertical axis represents density, which reflects the internal cohesion of the cluster, describing how strongly the terms within the cluster are interconnected [64]. This value illustrates the ability of a research theme to sustain itself and evolve over time [65].

The strategic diagram is divided into four quadrants. Themes in the upper right quadrant (Quadrant I) are considered motor themes, as they exhibit high centrality and density. Themes in the upper left quadrant (Quadrant II) are well-developed but relatively isolated, meaning they are not strongly connected to other themes. Themes in the lower left quadrant (Quadrant III) are less developed and may be emerging or declining in importance. Finally, themes in the lower right quadrant (Quadrant IV) are weakly structured, with low density, but have the potential to grow in significance due to their high centrality [66,67].

## 3. Results

This study undertook a comprehensive review of articles focusing on neuromodulation treatment for the language ability of patients with AD, published between 2006 and 2024. The analysis encompassed 88 pertinent studies disseminated across 65 outlets over the past 18 years. These studies were authored by 580 individuals, with an average citation count of 34.82 per document. The majority of authors, representing 99.31% (576 authors), engaged in collaborative endeavors, while a minority of 0.69% (4 authors) contributed to single-authored studies. The included studies employed varying criteria to diagnose Alzheimer’s disease. Most relied on established clinical diagnostic frameworks such as the NINCDS-ADRDA criteria or DSM-IV/DSM-5 guidelines, often based on cognitive and functional assessments. A subset of more recent studies supplemented clinical criteria with neuroimaging findings (e.g., amyloid PET and hippocampal atrophy on MRI) or cerebrospinal fluid biomarkers to support a biological diagnosis of AD, particularly in cases of atypical presentations such as LPA. However, explicit reporting of diagnostic criteria was inconsistent across studies, highlighting the need for standardized diagnostic reporting in future neuromodulation research involving AD populations. Therapeutic efficacy across the included studies was primarily evaluated using standardized clinical and behavioral instruments, such as the Boston Naming Test (BNT), the Alzheimer’s Disease Assessment Scale-Cognitive Subscale (ADAS-Cog), and various language-specific tasks including picture naming and auditory comprehension assessments. A limited number of studies incorporated neurophysiological measures—such as electroencephalography (EEG) and functional magnetic resonance imaging (fMRI)—to assess neuromodulatory effects on cortical activity. While biochemical indicators were occasionally reported, they did not constitute primary outcome measures in the majority of studies.

### 3.1. Standalone Research Domain

A noteworthy trend emerges from our review of the scientific literature on the application of neuromodulation to treat language impairment associated with AD. Specifically, 532 authors have only contributed one paper apiece, while one author has been involved in up to seven published studies. As to Pao’s results, Lotka’s law generally produces *β* values in the range of 1.78 to 3.78 in the majority of fields. The calculated *β* value of 2.95 from our analysis falls within Pao’s indicated range. Therefore, the utilization of neuromodulation in the treatment of AD-related language impairment can be considered a distinct research area, characterized by a remarkable concentration of authorship.

### 3.2. Publication Trend

**Figure 2:**
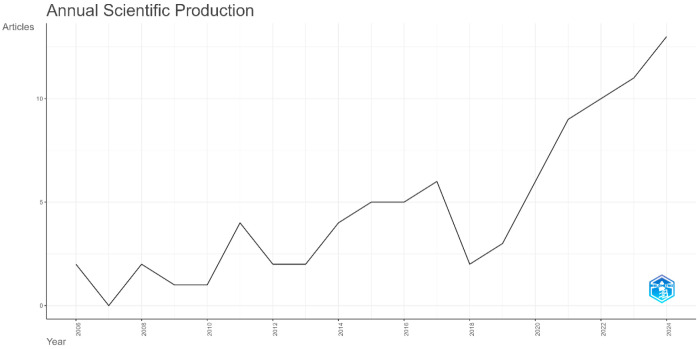
The yearly publication trends.

The annual trends in publications pertaining to neuromodulation techniques in AD’s language are depicted in Figure 2. The average growth rate for scientific research papers on neuromodulation treatment in AD’s language over the timespan from 2006 to 2024 was 10.96%. While the overall trend in the annual quantity of articles has demonstrated an increase over the 18-year period, the growth rate has exhibited variability over time. Given the significant decrease in pertinent studies in 2018, we examined the varied growth rate across three distinct time intervals: from 2006 to 2017, 2017 to 2018, and from 2018 to 2024. Between 2021 and 2024, there was a particularly noticeable increase in publications, accounting for 48.86% (43 out of 88) of all included articles. A total of 45 publications were produced in the first 15 years (2006–2021), which is nearly equivalent to the 43 publications produced in the last three years (2021–2024).

### 3.3. Publication Patterns

Based on the nations of the corresponding authors, Table 1 shows that the majority of the included articles were written by academics from ten different countries. Italy emerged as the largest contributor, accounting for 21.59% (19 out of 88) of the research, ahead of the US (20.46%) and China (17.05%). Table 2 shows that these articles were published in 65 journals, with the *Journal of Alzheimer’s Disease* having the highest percentage at 6.82%, followed by *Frontiers in Aging Neuroscience* (5.68%) and *Frontiers in Neurology* (4.55%).

In delineating the domains of inquiry encompassed within the field of neuromodulation-based AD treatment for language deficits, we made reference to the subject categories designated by WoS for the publications included in our analysis (Table 3). The analysis of research domains reveals a diverse distribution of publications, with neurosciences and neurology being the dominant domain, accounting for 44 publications, which represent half of the total dataset. Interdisciplinary research is also prominent, with nine publications (10.23%) linked to both psychology and neurosciences/neurology and six publications (6.82%) spanning geriatrics and gerontology and neurosciences and neurology. These intersections highlight the strong connection between neurological research, cognitive studies, and aging. Smaller research domain combinations, such as audiology and speech–language pathology, linguistics, and rehabilitation, appear in two publications, accounting for 2.27% of the total, indicating focused but important contributions in the field of communication disorders and rehabilitation. Overall, the concentration of 77.78% of publications in neuroscience-related domains underscores the central role of neuroscience in this research corpus. Meanwhile, the presence of smaller domains and interdisciplinary links highlights the growing trend toward cross-disciplinary collaboration, reflecting the complexity of neuromodulation treatment on language impairment in AD.

In order to identify the most impactful articles, we employed bibliometric measures, specifically TGC and TLC. Table 4 presents the most cited papers, ranked according to their TGC and TLC values. An analysis of the TGC and TLC criteria reveals that the most influential publications concerning the field of neuromodulation treatment for AD-related language impairment are those of Binney et al. (2010) [68] and Cotelli et al. (2011) [39], which achieved TGCs of 330 and TLCs of 29.

### 3.4. Academic Collaborations

**Figure 3:**
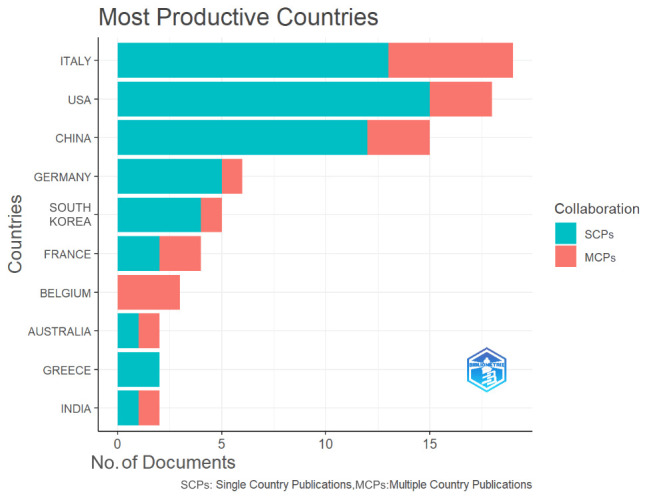
The most productive countries and the distribution of single or multiple country publications.

The nation/region-level scientific cooperation in neuromodulation therapy for AD-related language impairment was methodically examined and graphically shown in this bibliometric study. The analysis of collaborations among the top 10 most productive countries, as shown in Figure 3, distinguished between Single-Country Publications (SCPs) and Multiple-Country Publications (MCPs). The bars represent the number of documents produced by each country, while the colored sections show the proportion of SCPs (in turquoise) and MCPs (in red). The top contributing country, Italy, leads the field with 19 publications, of which a significant proportion are MCPs, reflecting Italy’s high level of international collaboration in this area. This is followed closely by the USA, which also demonstrates a strong output, particularly in MCPs, indicating the country’s extensive collaborative networks with other nations. Similarly, China, in third position, shows a high publication count, with a balanced distribution between SCPs and MCPs, highlighting its role both in independent research and in international cooperation. Germany and South Korea also contribute notable numbers of outputs, though their research activities are more concentrated in SCPs, suggesting a tendency toward domestic research efforts. In contrast, countries such as Australia, Greece, and India display smaller publication numbers, with higher proportions of MCP, signifying their reliance on international collaborations to advance research in this domain. Overall, the figure illustrates that while some countries, like Italy and the USA, maintain robust international collaboration networks, other nations tend to prioritize national research efforts. Despite this variation, the widespread presence of MCPs across most countries underscores the global nature of neuromodulation research in AD treatment, reflecting a concerted effort by nations to address this complex neurological challenge through shared scientific endeavors.

### 3.5. Key Research Themes

Keywords serve as fundamental indicators of research priorities within a given field. This study analyzed a total of 199 keywords, identifying the ten most frequently co-occurring terms, as shown in Table 5. The most prevalent keyword is “Alzheimer‘s disease” (23 occurrences), followed by “transcranial direct current stimulation” (15) and “stimulation” (12). The top keywords can be broadly categorized into four principal groups: neuromodulation techniques, cognitive functions, disease conditions, and research methodologies. This classification highlights the specific neuromodulation interventions applied, the cognitive impairments addressed, the medical conditions under investigation, and the methodological approaches employed. Notably, non-invasive brain stimulation methods, particularly transcranial direct current stimulation (tDCS) and repetitive transcranial magnetic stimulation (rTMS), emerge as focal points, underscoring a sustained research interest in these techniques for enhancing language and cognitive function in AD. Moreover, the presence of meta-analysis and systematic reviews among the most frequent keywords suggests a growing emphasis on evidence synthesis and methodological rigor in this field. These trends reflect an ongoing effort to establish scientifically validated, clinically effective neuromodulation strategies for AD-related language impairments.

**Figure 4:**
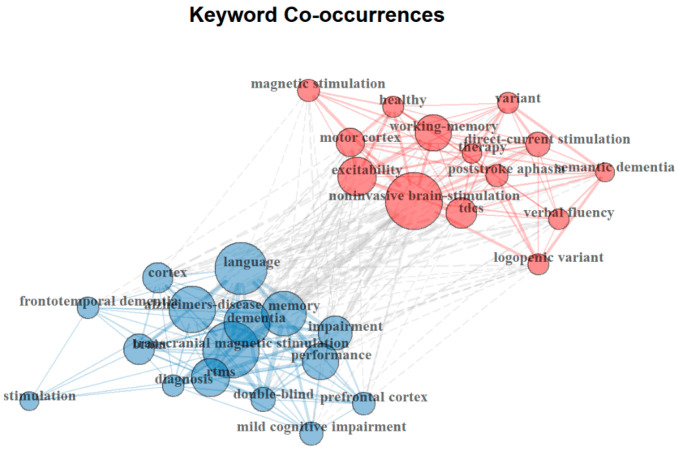
Research clusters identified based on the co-occurrence of authors’ keywords.

The co-word network analysis presented in Figure 4 illustrates the co-occurrence relationships among keywords in neuromodulation research for AD-related language impairments. In this network, each node represents a keyword, with node size reflecting its frequency of occurrence, while the thickness of the connecting lines indicates the strength of co-occurrence between keywords. The analysis identifies two major clusters, each representing a distinct research focus within the field.

The red cluster, centered around “noninvasive brain stimulation,” “direct-current stimulation (tDCS)”, and “excitability”, highlights a research focus on the neural mechanisms of neuromodulation and its therapeutic applications. This cluster emphasizes the role of cortical excitability, motor cortex activity, and working memory enhancement in neuromodulation-based interventions. Additionally, the inclusion of “post-stroke aphasia” and “semantic dementia” within this cluster suggests an extended interest in applying neuromodulation beyond AD, particularly in stroke-induced and progressive language disorders. The blue cluster, dominated by “Alzheimer’s disease”, “language”, “memory”, and “transcranial magnetic stimulation (TMS),” reflects a strong emphasis on the clinical applications of neuromodulation in AD and dementia-related cognitive impairments. This cluster underscores the increasing use of TMS and rTMS in treating memory and language deficits. The presence of terms such as “diagnosis”, “mild cognitive impairment”, and “double-blind” suggests a growing reliance on rigorous clinical trial methodologies to evaluate neuromodulation’s efficacy in AD therapy.

Despite their distinct focuses, cross-cluster connections indicate an increasing convergence between mechanistic research and clinical applications. Notably, the linkage between “prefrontal cortex” in the blue cluster and “motor cortex” in the red cluster suggests a shared interest in understanding cortical plasticity and neuromodulation’s role in cognitive restoration. Altogether, these findings highlight two dominant themes: (1) the investigation of neural mechanisms underlying brain stimulation techniques and (2) the application of neuromodulation to treat cognitive and linguistic impairments in AD and related conditions. The strong emphasis on noninvasive techniques like TMS and tDCS reflects their increasing role in clinical interventions. Moreover, the integration of experimental neuroscience and clinical research underscores an ongoing effort to translate neuromodulation from theoretical exploration into practical, evidence-based treatments for AD and associated language disorders.

**Figure 5:**
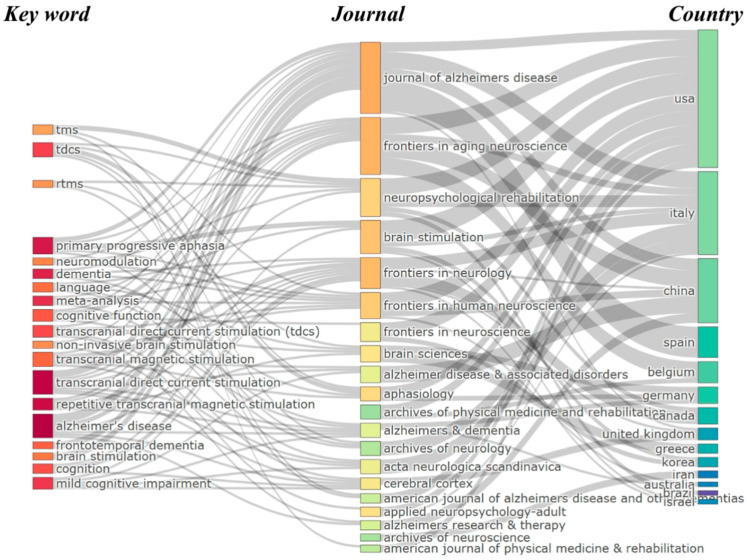
Three-field plot of the relationship among top publication sources, keywords, and countries.

Figure 5 presents a three-field plot illustrating the interconnections between key publication sources (left), author keywords (middle), and contributing countries (right) within the domain of neuromodulation treatments for language impairment in AD. The diagram, constructed using a Sankey visualization, reflects the flow and relationships between these three dimensions. The size of the boxes represents the frequency of occurrences, while the width of the connecting lines indicates the strength of the associations between them.

The leading journals in this field include the *Journal of Alzheimer’s Disease*, *Frontiers in Aging Neuroscience*, and *Neuropsychological Rehabilitation*, which serve as primary outlets for research on non-invasive neuromodulation techniques such as tDCS and rTMS. These journals facilitate the dissemination of studies investigating the impact of neuromodulation on cognitive and language impairments in AD. In terms of keywords, dominant research topics include tDCS, rTMS, TMS, neuromodulation, and AD, reflecting a strong emphasis on non-invasive brain stimulation for cognitive and language rehabilitation. Additionally, terms such as primary progressive aphasia and frontotemporal dementia suggest a growing interest in applying neuromodulation techniques beyond AD to other neurodegenerative conditions affecting language function. Regarding contributing countries, the USA, China, and Italy emerge as the most prolific nations in this research domain, with strong associations with both leading journals and core research themes. Other key contributors, such as Germany, Spain, Belgium, and the United Kingdom, also play significant roles in advancing research on brain stimulation and AD-related impairments. The presence of contributions from Canada, Australia, South Korea, and Iran further highlights the global nature of research in this field. This three-field plot effectively demonstrates the collaborative and interdisciplinary nature of neuromodulation research for AD, with key nations, prominent journals, and major research themes converging. The visualization underscores the continued growth and international engagement in advancing neuromodulation therapies, particularly in the development of non-invasive techniques such as tDCS and rTMS for treating language and cognitive impairments in AD.

### 3.6. Research Trends

**Figure 6:**
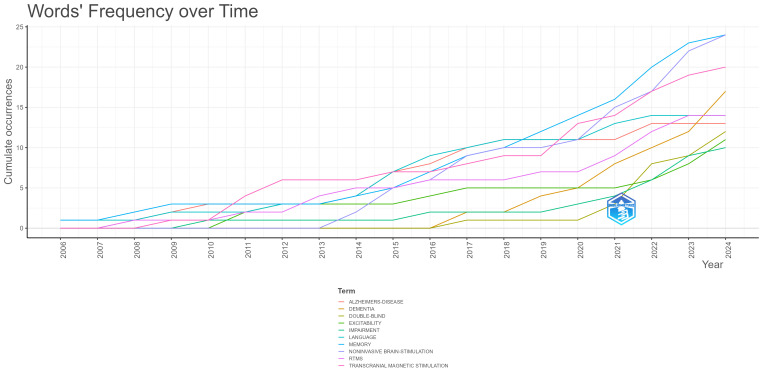
Word dynamics graph based on author keyword cumulative occurrences.

Figure 6 illustrates the cumulative occurrences of the top 10 keywords used by authors in neuromodulation treatment for language impairment in AD, spanning 2006 to 2024. All keywords exhibit a steady upward trend, with “Alzheimer’s disease” leading in cumulative occurrences, followed closely by keywords related to neuromodulation techniques, such as “transcranial direct current stimulation (tDCS)” and “repetitive transcranial magnetic stimulation (rTMS)”.

There has been a marked rise in the usage of tDCS and rTMS since 2015, reflecting growing interest and increased investigation into non-invasive neuromodulation techniques for treating AD-related language impairments. This surge highlights the expanding exploration of brain stimulation as a therapeutic avenue. Additionally, the use of keywords like cognition and cognitive function shows consistent growth, which aligns with the core focus on mitigating cognitive decline and restoring language capabilities in AD patients.

Keywords such as dementia and mild cognitive impairment also demonstrate moderate but steady growth, indicating a sustained focus on broader neurodegenerative conditions that contribute to AD-related language and cognitive difficulties. On the other hand, more specific conditions like primary progressive aphasia and frontotemporal dementia display a slower, yet consistent, increase, reflecting their relevance in more specialized studies of language impairment associated with AD.

In summary, the data points to an increasing concentration on neuromodulation techniques like tDCS and rTMS, with a clear focus on language and cognitive impairments in AD. This upward trajectory across all keywords reflects the growing momentum in research and clinical applications aimed at improving language function through neuromodulation in AD.

**Figure 7:**
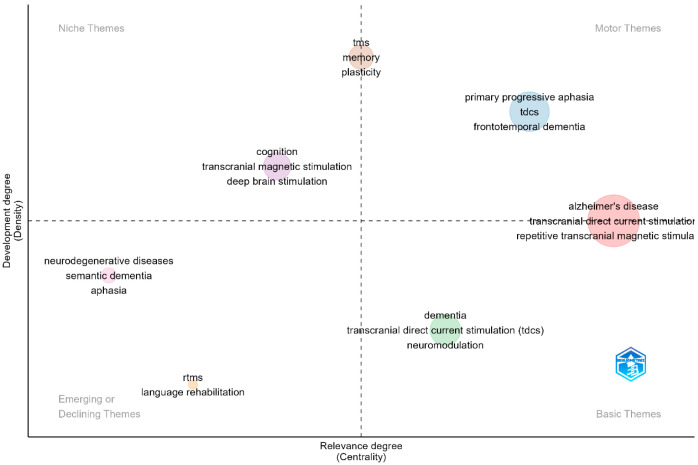
Key research themes in neuromodulation interventions for language deficits in Alzheimer’s disease.

For the thematic analysis investigating the relevance of topics based on their density and centrality, author keywords were selected as the unit of analysis. The analysis was performed on a set of keywords, identifying key research themes on the thematic map (Figure 7). These themes were categorized into clusters, with each cluster labeled by the most representative descriptors. Specifically, several clusters were positioned in the motor themes quadrant, indicating well-developed and central research topics, while others were distributed among the basic themes and emerging or declining themes quadrants.

In the motor themes quadrant (upper-right), which signifies topics with high centrality and density, important and well-established research themes are positioned. These themes demonstrate both significant interconnectivity with other topics and strong internal coherence, reflecting their maturity in the field of neuromodulation treatment for language impairments in AD. The dominant themes in this quadrant include “Alzheimer’s disease”, “transcranial direct current stimulation (tDCS)”, and “primary progressive aphasia”. These themes indicate that research on non-invasive neuromodulation interventions for cognitive and language deficits in AD is well-integrated and growing. The presence of “frontotemporal dementia” in this quadrant suggests that neuromodulation is increasingly being explored beyond AD, encompassing related neurodegenerative disorders affecting language function.

The basic themes quadrant (lower right) includes topics that are well-connected but still in the foundational stages of development. These themes are gaining traction within the research community, with increasing attention from scientists. “Dementia”, “neuromodulation”, and “tDCS”, are key examples within this quadrant, which indicates that while neuromodulation techniques are widely studied, their clinical applications in AD and related conditions are still evolving. The presence of “transcranial direct current stimulation (tDCS)” in both the basic and motor themes quadrants suggests that it is not only a fundamental concept in the field but also an actively expanding area of investigation.

The niche themes quadrant (upper left) contains highly developed but relatively isolated topics such as “memory”, “plasticity”, “deep brain stimulation”, and “transcranial magnetic stimulation”. These themes indicate specialized areas of research that, while significant, have fewer direct links to the broader field of neuromodulation for AD-related language deficits. The inclusion of “memory” and “plasticity” suggests a focus on understanding the neurophysiological mechanisms underlying neuromodulation interventions.

The emerging or declining themes quadrant (lower left) consists of topics that are either under early development or losing prominence in the field. This quadrant includes “language rehabilitation”, “repetitive transcranial magnetic stimulation (rTMS)”, “semantic dementia”, and “aphasia”. While these topics remain relevant, their placement suggests that more research integration is needed to strengthen their role in neuromodulation studies. The positioning of “language rehabilitation” in this quadrant highlights a potential gap in neuromodulation research, indicating that further studies are necessary to assess its efficacy in language recovery for AD patients.

**Figure 8:**
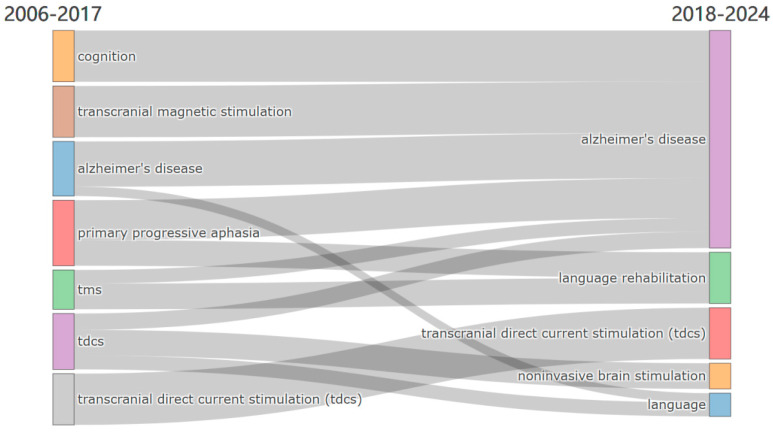
Evolution of main research themes from 2006–2017 to 2018–2024.

The thematic map (Figure 8) provides a structured visualization of the research landscape, demonstrating that neuromodulation for AD-related language impairments is an evolving field. The presence of well-established themes in the motor quadrant underscores the maturity of research on tDCS, rTMS, and AD, while the distribution of emerging and niche topics highlights areas of ongoing development and specialization. These findings suggest that while neuromodulation techniques are gaining traction in cognitive and language rehabilitation, further integration of emerging topics, particularly in language rehabilitation, is necessary to advance the field.

The thematic evolution from 2006 to 2024 is visualized through a Sankey Diagram (Figure 8), showcasing the development and continuity of key research topics over time. The analysis is divided into two distinct periods—2006–2017 and 2018–2024—providing insight into how certain themes have evolved and others have emerged within the field of neuromodulation treatment for AD and language impairments.

During the period from 2006 to 2017, research in neuromodulation treatments for language and cognitive impairments primarily focused on themes such as transcranial magnetic stimulation (TMS), Alzheimer’s disease (AD), cognition, primary progressive aphasia (PPA), and transcranial direct current stimulation (tDCS). These themes highlight an early emphasis on exploring neuromodulation as a potential intervention for neurodegenerative disorders affecting language and cognition, with AD emerging as a central research focus. The presence of PPA in this period suggests that researchers were particularly interested in targeting language deficits through neuromodulation techniques.

In the subsequent period (2018–2024), several themes have persisted and solidified their relevance, particularly AD and tDCS, which remain key focal points in the research landscape. Additionally, new themes such as language rehabilitation, noninvasive brain stimulation, and language have gained prominence, reflecting a shift toward broader therapeutic applications of neuromodulation. The continued emphasis on tDCS across both periods underscores its sustained significance as a non-invasive therapeutic approach for language and cognitive impairments in AD. Furthermore, the emergence of language rehabilitation as a distinct theme signals an increasing focus on clinical applications aimed at restoring communication abilities in affected individuals.

This thematic evolution illustrates the growing complexity and diversification of research in neuromodulation for AD-related language dysfunction. While foundational themes like AD and tDCS continue to dominate the field, the integration of new topics, such as noninvasive brain stimulation and language rehabilitation, suggests an expanding scope that encompasses both mechanistic investigations and translational applications. This progression underscores a heightened awareness of the multifaceted nature of AD-related cognitive and language impairments and the expanding role of neuromodulation in addressing these challenges.

## 4. Discussion

This study investigated 88 research articles on neuromodulation techniques for treating language impairments in AD published between 2006 and 2024. Utilizing bibliometric data, we analyzed publication trends, collaboration networks, and key research themes. We also identified the characteristics of global research activities, highlighting major contributors like Italy, the United States, and China, and explored emerging research hotspots. Additionally, this study examines the potential benefits of non-invasive and invasive neuromodulation techniques in enhancing language function in AD patients, while discussing the challenges and future directions in this rapidly evolving field.

### 4.1. Main Findings of Bibliometric Analysis

Since the initial studies emerged in 2006, research on neuromodulation techniques for treating language impairments in AD has gradually gained momentum. In particular, starting from 2021, the field has experienced a significant uptick in publications. Remarkably, almost half of the research articles in this area were published within the last three years, signaling a rapid expansion in interest. Several key factors have contributed to this recent growth, including advancements in non-invasive neuromodulation methods like TMS and tDCS [39,45]. These techniques have shown increasing potential in clinical applications, particularly for cognitive and language rehabilitation [39,42,43,47]. With this upward trend, it is likely that the volume of research on neuromodulation for AD-related language deficits will continue to expand in the near future.

Research on neuromodulation techniques for treating language impairments in AD is concentrated within neurosciences, neurology, and geriatric medicine journals, indicating a strong alignment with disciplines focused on neurological degeneration and cognitive rehabilitation [46]. This interdisciplinary research leverages cognitive neuroscience, speech-language pathology, and aging studies, which support the development of precise, clinically effective interventions. Key sources such as the *Journal of Alzheimer’s Disease* and *Frontiers in Aging Neuroscience* underscore the field’s rigorous methodological approach and focus on non-invasive methods, particularly TMS and tDCS. The prominence of peer-reviewed publications in these journals reveals the critical role of neuromodulation in managing AD-associated cognitive and language deficits. Moreover, contributions from cognitive psychology, neuropsychology, and speech therapy elucidate the broader cognitive and linguistic processes underlying AD, directly informing neuromodulation strategies. This integrative framework enhances the specificity and potential impact of neuromodulation interventions, advancing a robust, multifaceted approach to mitigating AD’s impact on language and communication abilities.

The field of neuromodulation for treating language impairments in AD has attracted considerable global interest, with collaborations emerging among researchers worldwide. Key contributors, including Italy, the United States, and China, have made significant strides; however, a substantial proportion of the research remains limited to single-country studies. This may stem from the medical and ethical complexities associated with neuromodulation interventions, particularly in vulnerable populations such as AD patients, which can make cross-national research initiatives more challenging [41]. Despite these challenges, expanding international collaborations in this field is critical. Multinational studies enable a more nuanced understanding of patient needs and clinical responses in varied healthcare systems, ensuring that neuromodulation therapies are safe, effective, and ethically grounded. Furthermore, global collaboration facilitates the exchange of diverse medical practices and ethical frameworks, thereby promoting the development of standardized protocols for neuromodulation interventions. By combining data across countries, researchers can increase the generalizability and impact of neuromodulation strategies, ultimately supporting more effective, globally relevant treatments for AD-related language impairments.

An analysis of the top keywords within the identified categories of neuromodulation research for AD-related language impairments highlights primary areas such as TMS, tDCS, memory, dementia, and language rehabilitation. Recent trends indicate a strong focus on non-invasive brain stimulation techniques like TMS and tDCS, reflecting their increasing application in clinical contexts to address AD-related cognitive and language deficits. This emphasis on non-invasive interventions underscores a shift toward developing accessible and targeted treatments for AD, which is crucial given the limitations of pharmacological approaches. Notably, research on PPA has emerged as a focal area, likely due to its direct association with AD pathology and the challenges it presents for diagnosis and therapy. Neuromodulation methods are proving valuable for developing individualized, condition-specific interventions in PPA, providing a basis for more tailored cognitive rehabilitation strategies [21,39]. Furthermore, these techniques support broader goals in AD research by offering a means to enhance cognitive functions such as memory and language, essential for improving quality of life in AD patients [45].

These findings suggest that the field of neuromodulation is evolving toward more specialized applications, utilizing advanced brain stimulation technologies to address specific cognitive impairments. This targeted approach not only broadens the therapeutic options for AD but also advances the development of precise, patient-centered treatments within neurodegenerative disease research.

### 4.2. Benefits of Applying Neuromodulation Techniques in the Treatment of Language Deficits in Alzheimer’s Disease

Neuromodulation holds significant promise in advancing our understanding of the pathological mechanisms underlying AD, especially as they pertain to language and cognitive decline. By targeting specific brain regions, focused stimulation helps identify the roles of these brain regions in processing language, elucidating the neural pathways and connections that degenerate in AD [16,17]. Understanding how neuromodulation influences neural activity in these areas offers critical insights into the disease’s progression, potentially pinpointing early markers of decline. Furthermore, emerging tools for early diagnosis, such as blood biomarkers (e.g., p-Tau217), are showing promise in detecting the disease prior to full clinical manifestation, which can aid in identifying patients who may benefit most from early neuromodulation interventions [69,70]. Moreover, neuromodulation sheds light on functional relationships within the broader brain networks involved in AD pathology, such as connectivity patterns between language-processing centers and memory-related areas, aiding in the discovery of new intervention points for disease management [16,18,20].

Additionally, neuromodulation presents clear benefits in targeted improvement of language and cognitive functions for AD patients. Many individuals with AD experience specific language impairments, including naming difficulties, sentence comprehension issues, and semantic processing deficits, which impede their ability to communicate and significantly reduce their quality of life [10,11,12,13]. Neuromodulation allows for the selective stimulation of language-related brain regions, directly supporting the neural activity required for effective language processing [18,20]. This targeted approach facilitates improvements in language fluency, comprehension, and semantic accuracy, enhancing communication abilities and allowing patients to maintain social connections. In contrast to general pharmacological treatments, which often lack specificity in addressing language deficits, neuromodulation offers a more precise intervention, focusing on the functional restoration of affected areas [5,7]. Notably, recent studies have extended stimulation targets beyond the traditional language network to include subcortical regions such as the cerebellum, which has shown relevance for language modulation in neurodegenerative conditions [71].

A particularly compelling aspect of neuromodulation’s therapeutic potential is its early intervention capacity in AD. Language deficits are often among the earliest indicators of cognitive decline in AD, manifesting well before severe cognitive symptoms emerge [5,7]. By intervening at this initial stage, neuromodulation may delay further deterioration, thereby extending patients’ cognitive and communicative functions. Early stimulation of language-relevant regions has the potential to strengthen neural pathways, reinforcing the brain’s capacity to compensate for areas of damage [37,39,42,43]. This proactive approach aligns with modern trends in preventative medicine, where early interventions aim to modify disease trajectories before significant impairment occurs. Enhancing neural plasticity and cognitive resilience through early neuromodulation not only supports language abilities but may also contribute to an overall slowing of disease progression [38,45,47].

Furthermore, neuromodulation’s benefits extend to personalized treatment plans for patients. Neuromodulation’s adaptable parameters (i.e., frequency, intensity, and duration) allow clinicians to tailor treatment to each patient’s specific language and cognitive deficits, offering a flexible, patient-centered approach that aligns with the growing emphasis on precision medicine in AD care [20,72]. This customized therapy not only enhances outcomes but also minimizes the need for intensive and generalized cognitive interventions, reducing the frequency of hospital visits and lowering the strain on healthcare resources. Given the diagnostic overlap between AD and language-variant frontotemporal dementia (FTD), particularly in early stages, individualized neuromodulation strategies must be grounded in accurate differential diagnosis. Multimodal approaches—combining structural imaging, molecular biomarkers, and cognitive profiling—are essential to distinguish AD-related logopenic PPA from FTD subtypes and to guide appropriate therapeutic targeting [14,15]. Such stratification enhances the efficacy and specificity of interventions, thereby facilitating clinical decision-making in the context of diagnostic uncertainty.

Deep brain structures, particularly the thalamus and basal ganglia, play a fundamental role in language processing by maintaining stable semantic representations and coordinating with cortical regions involved in lexical, syntactic, and semantic functions [73,74]. In AD, the progressive degeneration of these subcortical circuits disrupts cortico–thalamo–cortical communication, leading to impairments in word retrieval, comprehension, and overall linguistic fluency [73]. Neuromodulation techniques like DBS have shown substantial success in managing motor symptoms in treating movement disorders, particularly PD. The expansion of neuromodulation to treat AD—specifically targeting language deficits—further validates its versatility and clinical applicability, underscoring neuromodulation’s potential beyond movement disorders [32,75]. This successful application in AD not only strengthens neuromodulation’s credibility in the medical community but also highlights its adaptability in addressing diverse neurological impairments and encourages broader acceptance and integration of these techniques in routine medical practice, paving the way for the development of neuromodulation techniques and providing better rehabilitation plans for patients.

### 4.3. Challenges and Future Developments

The incorporation of neuromodulation techniques into the treatment of AD-related language deficits introduces complex challenges that require careful consideration. A primary concern involves the ethical implications of employing neuromodulation to modulate linguistic functions in AD patients [76,77]. Thoughtful ethical oversight is essential to ensure that these interventions prioritize patient well-being and respect individual autonomy in therapeutic contexts. To ensure responsible implementation, ethical frameworks must evolve in tandem with advancements in neuromodulation technologies. These frameworks should address complexities such as assessing decision-making capacity in AD patients and involving caregivers as critical partners in treatment planning and monitoring. Moreover, the maintenance of neuromodulation devices necessitates long-term strategies that prioritize both safety and efficacy, involving comprehensive support systems for patients and their families. Also, establishing robust ethical boundaries is essential to prevent over-reliance or misuse of neuromodulation as its clinical applications expand. Future efforts must also prioritize research into the long-term effects of neuromodulation on autonomy, quality of life, and the broader implications for its use in neurodegenerative conditions, ultimately creating a sustainable and ethically sound roadmap for its continued development.

Building on the ethical considerations surrounding the application of neuromodulation, another important aspect to address is the potential risks these techniques pose to patient health, particularly given the vulnerability of individuals with AD. Neuromodulation treatments involve the application of electrical or magnetic stimulation to specific brain regions, which may lead to unintended adverse effects. Previous studies have reported adverse effects including transient headaches, scalp discomfort, dizziness, and in rare cases, seizures, especially when applied inappropriately or without adhering to established safety protocols [78,79,80]. Furthermore, the complexity of AD pathology introduces further challenges. AD patients often experience vascular comorbidities [81,82] or brain atrophy [83,84], which can alter the safety profile of neuromodulation. This variability raises concerns about the unpredictability of treatment effects and underscores the importance of individualized treatment planning. Also, while short-term improvements in cognitive and language functions have been consistently observed, the safety of frequent or prolonged neuromodulation use is not yet fully established. Improper application or overuse may lead to unintended consequences, such as neural fatigue, maladaptive plasticity, or other adverse effects that could compromise, rather than enhance, cognitive function.

To address these issues concerning patients’ safety and potential risks, it is imperative to conduct comprehensive patient assessments prior to initiating neuromodulation therapy. Evaluations should include a detailed analysis of individual risk factors, such as comorbid conditions and structural brain abnormalities, to tailor treatments and mitigate potential harm. In addition, rigorous monitoring during and after therapy is essential for the early detection and management of any adverse effects, ensuring patient safety throughout the course of treatment. Future research should prioritize the refinement of neuromodulation protocols to enhance their precision and efficacy. Efforts should focus on improving targeting accuracy, optimizing treatment parameters, and identifying reliable biomarkers to predict patient responses and assess long-term safety outcomes. Such advancements are critical to ensuring that the therapeutic benefits of neuromodulation outweigh its risks, ultimately providing safe and effective interventions for individuals with AD.

A major challenge in widely applying neuromodulation techniques for treating AD-related language deficits lies in the stringent requirements for clinical conditions. Most neuromodulation procedures necessitate a controlled medical environment equipped with advanced imaging and support systems to support precise and sterile procedures and ensure patient safety. This requirement is particularly critical due to the potential for adverse effects, which, although infrequent, demand immediate medical intervention to mitigate serious risks effectively. In addition, the procedure must be performed by a multidisciplinary team, including a clinical neurophysiologist to monitor intraoperative brain activity and ensure accurate targeting of brain regions, a trained operator to configure stimulation parameters accurately, a trained physician or nurse to provide immediate care during emergencies, safeguarding patient well-being, and a neurosurgeon to implant the electrodes when conducting invasive therapies. These resource-intensive requirements create significant logistical and financial barriers, limiting the widespread implementation of neuromodulation techniques. Healthcare facilities in resource-limited or underserved areas often lack the necessary infrastructure, surgical capabilities, or adequately trained personnel to administer these treatments safely. Consequently, access to these advanced neuromodulation techniques remains restricted, particularly for patients in low-resource settings, exacerbating healthcare disparities.

Despite significant advancements in neuromodulation for AD-related language deficits, current techniques face critical limitations in effectively targeting deep brain structures. Non-invasive approaches, such as TMS and tDCS, are restricted to cortical stimulation, limiting their impact on subcortical networks that are integral to language processing and cognitive function [73,74]. In contrast, DBS can directly modulate these deeper neural circuits but remains an invasive procedure, presenting inherent surgical risks and long-term maintenance challenges [27]. This technological gap highlights a fundamental challenge in the field—there is currently no widely adopted neuromodulation technique capable of non-invasively reaching and modulating deep brain regions while ensuring clinical feasibility and patient safety. Addressing this limitation is essential for advancing neuromodulation as a viable therapeutic strategy for AD, ensuring that interventions can comprehensively target both cortical and subcortical regions involved in language processing and cognitive function.

Overcoming the challenges associated with neuromodulation techniques requires a focus on optimizing their implementation and accessibility. For non-invasive methods, the development of portable, user-friendly devices that adhere to strict safety standards can significantly enhance their usability across a variety of clinical settings. For surgical interventions, progress in automation and miniaturization, alongside increased training opportunities for healthcare professionals, can reduce dependence on specialized, resource-intensive facilities and expand the availability of these treatments. Future innovations, such as temporal interference (TI) stimulation, have shown promise in enabling precise, non-invasive modulation of subcortical structures, potentially overcoming the current dichotomy between invasiveness and effectiveness [85]. TI enables the non-invasive and precise stimulation of deep brain structures by employing intersecting high-frequency electrical currents to produce low-frequency stimulation at specific depths [85]. This breakthrough not only expands the scope of neuromodulation applications but also reduces the risks inherent in traditional invasive procedures, offering a safer and more versatile alternative for patients. Maintaining rigorous operational protocols remains a cornerstone for ensuring the safety and efficacy of neuromodulation in treating AD-related language deficits. Continued advancements in technology, such as the refinement of TI, combined with strategic resource allocation, will be crucial in overcoming existing barriers. Addressing these challenges will pave the way for wider clinical adoption of neuromodulation techniques, improving therapeutic outcomes and expanding treatment options for neurodegenerative disorders.

### 4.4. Limitations

This bibliometric review has several limitations that should be noted. First, it provides a broad overview of the global research landscape on neuromodulation for AD-related language deficits, rather than conducting a detailed analysis of specific techniques or their comparative efficacy. Future research could focus on individual methods to explore their specific contributions to language rehabilitation in greater depth. Second, the analysis was restricted to articles indexed in the Web of Science Core Collection, potentially omitting relevant studies from other databases. Expanding the scope to include publications in various languages and indexing platforms would provide a more globally representative perspective. Finally, while bibliometric analysis highlights trends and collaboration networks, it does not assess the quality or clinical impact of individual studies. Future research could integrate bibliometric approaches with systematic reviews or meta-analyses to provide a more comprehensive understanding of neuromodulation’s clinical applications for AD-related language deficits.

## 5. Conclusions

This literature review provides a comprehensive bibliometric analysis of neuromodulation research targeting AD-related language deficits, highlighting key contributors, global collaboration networks, and emerging research themes. Neuromodulation techniques, including rTMS, tDCS, DBS, and emerging methods like TI, demonstrate significant potential in addressing the cognitive and communicative challenges posed by AD. Our findings reveal rapid growth in this field and advances in non-invasive approaches, while also identifying critical challenges such as ethical considerations, resource-intensive operational requirements, barriers to clinical implementation, and improvements needed in existing techniques.

Although this review primarily adopts a bibliometric approach, the surveyed literature consistently reports encouraging evidence regarding the efficacy of neuromodulation interventions—particularly rTMS and tDCS—in improving naming, comprehension, and semantic processing in individuals with AD. DBS, while more invasive, has also shown potential benefits in modulating deep brain circuits involved in language functions.

By addressing these challenges and fostering interdisciplinary collaboration, this study offers a roadmap for advancing the field of neuromodulation. Future research should prioritize the refinement of neuromodulation protocols, the integration of novel techniques, and the expansion of access to ensure equitable and effective treatments. These efforts are essential for optimizing therapeutic outcomes and bridging the gap between research advancements and clinical practice. Ultimately, this work contributes to enhancing the quality of life for individuals with AD and furthering the integration of neuromodulation into comprehensive care strategies for neurodegenerative disorders.

## Figures and Tables

**Table 1:** The top 10 (by quantity/total citations) countries in neuromodulation interventions for language deficits in AD.

Rank	Country (Ranking by Output)	Output * *n* (%)	Country (Ranking by Citations)	Citations ** *n* (%)
1	Italy	19 (21.59)	Italy	1114 (36.37)
2	USA	18 (20.46)	USA	465 (15.18)
3	China	15 (17.05)	United Kingdom	361 (11.78)
4	Germany	6 (6.82)	China	257 (8.39)
5	South Korea	5 (5.68)	Belgium	242 (7.90)
6	France	4 (4.55)	South Korea	234 (7.64)
7	Belgium	3 (3.41)	Australia	169 (5.52)
8	Australia	2 (2.27)	Brazil	73 (2.38)
9	Greece	2 (2.27)	France	30 (0.98)
10	India	2 (2.27)	Germany	29 (0.95)

Note: A publication may have multiple corresponding authors. * N = 88, ** N = 3064.

**Table 2:** The top 10 (by quantity/total citations) journals/conferences in neuromodulation interventions for language deficits in AD.

Rank	Journals (Ranking by Output)	Output * *n* (%)	Journals (Ranking by Citations)	Citations ** *n* (%)
1	*Journal* *of Alzheimer’s Disease*	6 (6.82)	*Neurology*	233 (3.78)
2	*Frontiers in Aging Neuroscience*	5 (5.68)	*Brain Stimulation*	228 (3.69)
3	*Frontiers in Neurology*	4 (4.55)	*Brain*	194 (3.14)
4	*Frontiers in Human Neuroscience*	3 (3.53)	*Clinical Neurophysiology*	176 (2.85)
5	*Brain Stimulation*	3 (3.53)	*Neuroimage*	152 (2.46)
6	*Neuropsychological Rehabilitation*	3 (3.53)	*Journal of Neurology, Neurosurgery, and Psychiatry*	119 (1.93)
7	*Applied Neuropsychology-Adult*	2 (2.27)	*Journal of Alzheimer’s Disease*	116 (1.88)
8	*Cerebral Cortex*	2 (2.27)	*Neuropsychologia*	112 (1.81)
9	*Aphasiology*	2 (2.27)	*Annual Neurology*	104 (1.69)
10	*Brain Sciences*	2 (2.27)	*Journal of Neuroscience*	103 (1.67)

Note: * N = 88, ** N = 6172.

**Table 3:** The top 5 (by quantity/total citations) research domains.

Rank	Research Domains (Ranking by Output)	Output * *n* (%)	Research Domains (Ranking by Citations)	Citations ** *n* (%)
1	Neurosciences and Neurology	44 (50.00)	Neurosciences and Neurology	1945 (63.48)
2	Neurosciences and Neurology; Psychology	9 (10.23)	Neurosciences and Neurology; Psychiatry; Surgery	246 (8.03)
3	Geriatrics and Gerontology; Neurosciences and Neurology	6 (6.82)	Neurosciences and Neurology; Psychology	216 (7.05)
4	Audiology and Speech–Language Pathology; Linguistics; Neurosciences and Neurology; Rehabilitation	2 (2.27)	Geriatrics and Gerontology; Neurosciences and Neurology	109 (3.56)
5	Behavioral Sciences; Neurosciences and Neurology	2 (2.27)	Oncology; Cell Biology	100 (3.26)
5	Psychiatry	2 (2.27)	Rehabilitation; Sport Sciences	96 (3.13)

Note: * N = 88, ** N = 3064.

**Table 4:** The top 10 (by global/local citation counts per year) publications.

Rank	Article (Ranking by TGC)	Type	TGC	TGC/y	Article (Ranking by TLC)	Type	TLC
1	Binney et al., 2010	Original research	330	23.57	Cotelli et al., 2011	Original research	29
2	Van Overwalle et al., 2020	Review	225	56.25	Cotelli et al., 2008	Original research	20
3	Cotelli et al., 2011	Original research	216	16.62	Cotelli et al., 20 14	Original research	20
4	Cotelli et al., 2008	Original research	191	11.94	Cotelli et al., 20 06	Original research	19
5	Cotelli et al., 2006	Original research	166	9.22	Tsapkini et al., 2014	Original research	15
6	Meinzer et al., 2015	Original research	137	15.22	Trebbastoni, 2013	Original research	10
7	Lee et al., 2016	Original research	113	14.13	Hung et al., 2017	Original research	10
8	Im et al., 2019	Original research	104	20.80	Zhao et al., 2017	Original research	10
9	Zhao et al., 2017	Original research	100	14.29	Meinzer et al., 2015	Original research	9
10	Di Lazzaro et al., 2021	Review	96	32.00	Devi et al., 2014	Original research	8

Note: TGC: Total Global Citations; TLC: Total Local Citations; y: year.

**Table 5:** The top keywords relating to neuromodulation techniques and AD-related language impairment.

Rank	Keywords (Ranking by Occurrences)	Category	Occurrences
1	Alzheimer’s disease	disease	23
2	Transcranial direct current stimulation	Technique	15
3	Stimulation	Technique	12
4	Dementia	Disease	10
5	Primary progressive aphasia	Disease	9
6	Meta-analysis	Methodology	8
7	Repetitive transcranial magnetic stimulation	Technique	8
8	TDCS	Technique	8
9	Cognition	Function	7
10	Cognitive function	Function	7

## Data Availability

Publicly available datasets were analyzed in this study. This data can be found here: https://osf.io/t9er8/ (accessed on 18 March 2025).
